# The History of St. George's Hospital

**Published:** 1914-05-16

**Authors:** 


### THE HISTORY OF ST. GEORGE'S HOSPITAL.

### Some Eighteenth Century Institutional Officers.

With commendable celerity the "History of St. George's Hospital," which had been delayed through the illness of the author, Mr. G. C. Peachey, is being proceeded with now that publication of the parts has been resumed. The latest addition to the sections already published is, like the preceding one, a double number, and carries the work half-way.* It deals practically exclusively with the organisation and administration of the hospital during the early years after its foundation, and contains many interesting biographical details concerning the members of the medical staff in the first half of the eighteenth century. These latter biographies are compiled with great care and are a species of research wherein great labour is often represented by slender results. It behoves us, therefore, at once to acknowledge the complete and painstaking character of this section of the work: less showy perhaps than other chapters, but equally valuable and scholarly. In passing we would add that researches of the same kind into the lives of bygone worthies have been lately published in the magazine of another great London medical school.

### The First Secretary.

Of greater general interest are the earlier chapters of the present instalment of Mr. Peachey's history; for therein are given many most interesting details of hospital control and administration in the eighteenth century. In its early years St. George's was most unfortunate in its secretarial appointments. The first secretary was a Mr. Thomas Smith, who was a subscriber and undertook the office without fee or reward; this was in 1733. Two years later a writer was engaged to help the honorary secretary in posting the minutes. Before 1738 Mr. Smith died, and in that year a permanent clerk was appointed in his place. This was a Mr. Cade, who was paid a salary of £20 a year "and his dyet in the house." In 1740 this first professional secretary died, and Mr. Mason was elected, at the same salary, with a gratuity in addition. This, the third secretary, did not hold office long, for in 1743 he embezzled some of the hospital funds and was dismissed. The fourth secretary was Mr. Van Riell; but he absconded in 1745. Mr. Say was then appointed, and was made to give security for £100. He held office until his death in 1756. Thus in twenty-three years the hospital was served by five secretaries, three of whom died and two were dishonest. The institutional world has set its house in order since those days!

### Early Treasurers and Chaplains.

The first treasurers of the hospital were Captain Hudson and Mr. Aspinwall. They were, of course, honorary officers; after twelve years of service, oddly enough, the latter gentleman met with misfortunes, and a subscription was promoted to assist him. Bequests came in quite early in the history of the institution; one of the first consisted of some Bond Street house property, which is still in the possession of the charity, and was bequeathed by a Mr. Wandsell two years after the hospital was founded. Another interesting early gift (1733) was the chalice and patten, still in use in the hospital chapel, which were bought by the daughter of Sir Nathaniel and Lady Curzon out of a fund of £100 which the latter had left by will to be distributed according to her daughter's discretion. These utensils are of silver-gilt; they are of French manufacture, but the date seems a little uncertain, though it is anterior to 1733. The first formal appointment of a chaplain was in 1738; but Mr. Fraser, who was elected to the post, had already been officiating in an honorary capacity for three years. There was at this time no chapel within the buildings, but a part of one of the wards was reserved for the purpose; in 1744 one of the trustees bore the expense of building a separate chapel. This was, of course, not the present chapel, which is of more modern date.

### Political Prejudice and Professional Success.

An excellent sketch is given of the complicated evolutionary processes which resulted in the establishment in the eighteenth century of the three orders of practitioners—physicians, surgeons, and apothecaries. Political prejudices had a great deal to do with professional success in the higher ranks of the profession (i.e. at that time, the physicians); and it is interesting also to note that politics were allowed to dominate entire institutions. Thus St. Thomas's and Guy's were "run" on Whig principles, whereas St. Bartholomew's and Bedlam were Tory. St. George's, we gather, was free from these political influences; but was subject to religious influences, to the extent that it "was dominated by Protestant principles." There was, moreover, a religious test for those who were, or desired to be, connected with the institution in any administrative or menial capacity.

In those early days the visiting staff held their appointments for much shorter average periods than do their successors of to-day. Thus six physicians were appointed at the foundation of the charity in 1733; yet four of them resigned within three years, one served twelve years, and one twenty-four years. Seven more appointments were made to the medical staff before the hospital was seventeen years old; and the surgical staff during the same period comprised almost as many members. Of these old-time worthies Mr. Peachey gives many interesting particulars; and indeed Parts V. and VI. of his history are packed as full of institutional and antiquarian lore as those which have already appeared.

* *The History of St. George's Hospital.* By G. C. Peachey. Parts V. and VI. Price 5s. net. Printed for the Author by Bemrose and Sons.

